# Clinical features of oral lichen planus and oral lichenoid lesions: an oral pathologist’s perspective

**DOI:** 10.1590/0103-6440202204426

**Published:** 2022-06-24

**Authors:** Juliana de Noronha Santos Netto, Fábio Ramoa Pires, Karen Hurtado Andrade Costa, Ricardo Guimarães Fischer

**Affiliations:** 1 Post-graduation Program in Dentistry, Dental School, Rio de Janeiro State University, Rio de Janeiro/RJ, Brazil; 2 Oral Pathology, Dental School, Rio de Janeiro State University, Rio de Janeiro/RJ, Brazil; 3 Dental School, Rio de Janeiro State University, Rio de Janeiro/RJ, Brazil; 4 Periodontics, Dental School, Rio de Janeiro State University, Rio de Janeiro/RJ, Brazil

**Keywords:** Lichen planus, lichenoid lesions, oral mucosa, mouth

## Abstract

The clinicopathological features that precisely characterize oral lichen planus (OLP) and oral lichenoid lesions (OLL) still represent a challenge. The aim of the present study was to analyze, from an oral pathologist perspective, the clinical features from OLP and OLL. Specimens fullfilling the histological criteria for OLP and OLL, and also compatible with OLP (OLP-C), were selected and clinical information was retrieved from the laboratory forms. The final sample was composed by 221 cases, including 119 OLP (53.8%), 65 OLP-C (29.4%) and 37 OLL (16.7%). Females were more affected in the three groups, but the number of males was higher in OLL. Mean age was lower in OLP (52.3 years) in comparison with OLL (57.9 years) (p=0.020). Buccal mucosa and tongue involvement was more frequent in OLP; gingival involvement was uncommon in OLL. The reticular pattern was more frequently found in OLP, while the association of reticular and atrophic/erosive/ulcerated patterns was more common in OLP-C and OLL (p=0.025). In conclusion, gender and mean age of the patients, and anatomical location and clinical manifestation of OLL are different from OLP, and could help to better characterize this group of conditions. Specimens diagnosed as OLP-C showed clinical parameters close to OLP.

## Resumo

Os achados clinico-patológicos que caracterizam de forma precisa o líquen plano oral (LPO) e as lesões liquenoides orais (LLO) ainda representam um desafio. O objetivo do presente estudo foi analisar, pela perspectiva do patologista oral, os achados clínicos do LPO e das LLO. Espécimes preenchendo os critérios histológicos para o diagnóstico de LPO e LLO, e também aqueles compatíveis com LPO (LPO-C), foram selecionados e as informações clínicas foram obtidas a partir das requisições laboratoriais. A amostra final foi composta de 221 casos, incluindo 119 LPO (53.8%), 65 LPO-C (29.4%) e 37 LLO (16.7%). As mulheres foram mais acometidas nos três grupos, mas o número de homens acometidos foi maior nas LLO. A média de idade foi menor no LPO (52.3 anos) em comparação com as LLO (57.9 anos) (p=0.020). O envolvimento da mucosa jugal e da língua foi mais frequente no LPO; o envolvimento gingival foi incomum nas LLO. O padrão reticular foi mais frequentemente encontrado no LPO, enquanto a associação dos padrões reticular e atrófico/erosivo/ulcerado foi mais comum no LPO-C e nas LLO (p=0.025). Em conclusão, o gênero e a média de idade dos pacientes, assim como a localização anatômica e a manifestação clínica das LLO são diferentes do LPO, e podem ajudar a melhor caracterizar este grupo de condições. Espécimes diagnosticados como LPO-C mostraram parâmetros clínicos mais próximos ao LPO.

## Introduction

Oral lichen planus (OLP) is a chronic mucocutaneous inflammatory disease mediated by a T-cell immunomediated reaction ^1^ ^,^ ^2^. OLP affects about 1% of the general population, and most patients are females in their fifities to sixties ^1^ ^,^ ^2^. There are six clinical patterns of presentation of the disease: reticular, papular, plaque-like (white lesions), atrophic, erosive/ulcerative and bullous (red lesions) ^1^ ^,^ ^2^ ^,^ ^3^. The most common oral affected site is the buccal mucosa, followed by the tongue and the gingiva/alveolar mucosa, and lesions are usually bilateral and symmetric ^1^ ^,^ ^2^. Symptoms are present in about two thirds of the affected patients and usually include oral soreness, burning sensation and pain ^2^ ^,^ ^4^. Apart from oral involvement, patients affected by OLP can present lesions in other regions such as the skin, hair, nails, and genital region ^2^ ^,^ ^5^.

It is presently accepted that OLP diagnosis should be confirmed considering both clinical aspects of the lesions and histological analysis of biopsied tissues, the latter following some histological diagnostic criteria ^2^ ^,^ ^6^. The most typical histological features of OLP are a well-defined band-like zone of chronic inflammatory infiltrate composed mostly by lymphocytes confined to the basement membrane and superficial lamina propria, liquefaction of the basal cell layer and epithelial exocytosis ^2^ ^,^ ^6^ ^,^ ^7^. There are other conditions, however, that can clinically and histologically resemble OLP showing a considerable overlap in diagnostic criteria ^1^ ^,^ ^3^ ^,^ ^4^. These entities include mostly oral lichenoid lesions (OLL - mostly in response to local contact with restorative materials and other substances, and reactions to systemic administered drugs), graft versus host disease, chronic ulcerative stomatitis and lupus erythematosus ^1^ ^,^ ^2^ ^,^ ^3^ ^,^ ^8^ ^,^ ^9^. Furthermore, it is not uncommon for oral pathologists to come across with histological features showing a lichenoid pattern without fullfilling all microscopic criteria for OLP or OLL, rendering a report suggestive of/compatible with OLP (OLP-C) or lichenoid mucositis with no additional specification.

The first attempt to clinically and histologically characterize OLP was suggested in 1978 by the World Health Organization ^7^. A modification to this proposal was suggested by van der Meij and van der Waal in 2003 ^6^ and, more recently, Cheng et al. ^2^, representing a position of the American Academy of Oral and Maxillofacial Pathology, suggested some additional modifications. Even so, the essential features to characterize OLP and the clinical and histological criteria to consistently diagnose OLL still represent a challenge in both clinical and histological settings ^4^ ^,^ ^10^.

As biopsies are routinely indicated in the diagnostic proccess of OLP, but sometimes did not show all the histological criteria necessary for its diagnosis, oral pathologists are frequently challenged in this scenario. Therefore, the aim of the present study was to comparatively analyze the clinical features from a series of histologically specimens fullfilling the microscopic criteria for OLP and OLL.

## Materials and Methods

This is a retrospective descriptive and comparative study. All specimens that histologically fullfilled all criteria necessary for the diagnosis of OLP and OLL, and also all specimens with histological features compatible with OLP (OLP-C), were retrieved from the files of an Oral Pathology Laboratory from 2005 to 2019. After this initial review, specimens presenting no adequate/representative histological sections and/or paraffin blocks for eventual additional sections, presenting no complete clinical information, and diagnosed as graft versus host disease were excluded from the final sample. All specimens showing a lichenoid inflammatory infiltrate but diagnosed as any other condition based on the association with clinical parameters (e.g. some oral leukoplakias and leukoerythroplakias, and lupus erythematosus) and/or immunohistochemical/immunofluorescence findings (e. g. chronic ulcerative mucositis and lupus erythematosus), were also excluded from the final sample.

After this initial selection, all hematoxylin and eosin (HE) stained sections were reviewed and additional HE-stained sections were obtained when necessary. The histological criteria used for diagnosing OLP was strictly based on the proposed criteria suggested by Cheng et al. ^2^ and by Van der Meij and Van der Waal ^6^. Specimens diagnosed as OLL showed a band-like predominantly lymphocytic infiltrate in the lamina propria and also a deep infiltrate mostly arranged in a perivascular pattern and sometimes forming germinal centers. Histological sections presenting most, but not all, microscopic lichenoid features and absence of (or only presence of focal) deep lymphocytic inflammatory infiltrate without germinal centers were diagnosed as OLP-C.

Laboratory registries and biopsy submission forms were reviewed and all clinical information was obtained, including patient information (age and gender) and information on the clinical presentation of the disease (site distribution, site of biopsy, clinical aspect, onset of the disease, and symptoms). All information was descriptively and comparatively analyzed using the software Statistical Program for Social Sciences (version 2.0), with a significance level of 5% (p=0.05).

The study was conducted according to the World Medical Association Declaration of Helsinki and was approved by the local Ethics in Research Committee (number 3.770.736).

## Results

The final sample was composed by 221 patients, including 119 cases presenting all histological criteria for diagnosis of OLP (53.8%), 37 cases presenting the histological criteria for diagnosis of OLL (16.7%) and 65 cases characterized as OLP-C (29.4%). There were 160 females (72.4%) and 61 males (27.6%) (female:male ratio of 2.6:1) and mean age of the affected patients was 54 years (ranging from 16 to 86 years). Females were more affected in the three groups but in OLL the female:male ratio was lower (1.4:1) than in OLP (3.3:1) and in OLP-C (2.6:1) (Table 1). Mean age was lower in OLP (52.3 years) in comparison with OLL (57.9 years) (p=0.020), and no statistically significant differences were found when comparing with mean age of OLP-C (55.2 years).

**Table 1:** Distribution of the clinical parameters by histological diagnosis.

Parameter	Histological diagnosis (n=221) ^†^			P *
	OLP (n=119)	OLP-C (n=65)	OLL (n=37)	
Gender					
Female	91 (76%)	47 (72%)	22 (59%)	0.130
Male	28 (24%)	18 (28%)	15 (41%)	
Anatomical location					
Buccal mucosa				0.084
No	27 (23%)	24 (37%)	13 (35%)	
Yes	92 (77%)	41 (63%)	24 (65%)	
Tongue				0.465
No	61 (51%)	37 (57%)	23 (62%)	
Yes	58 (49%)	28 (43%)	14 (38%)	
Gingiva				0.141
No	92 (77%)	51 (78%)	34 (92%)	
Yes	27 (23%)	14 (22%)	3 (8%)	
Number of affected sites				0.108
One	54 (45%)	38 (58%)	27 (73%)	
Two	51 (43%)	20 (31%)	8 (22%)	
Three	9 (8%)	4 (6%)	2 (5%)	
More than three	5 (4%)	3 (5%)	-	
Clinical aspect				0.025
Reticular	73 (61%)	28 (43%)	16 (43%)	
Atrophic/erosive/ulcerated	4 (4%)	7 (11%)	1 (3%)	
Reticular + atrophic/erosive/ulcerated	42 (35%)	30 (46%)	20 (54%)	

† OLP - oral lichen planus; OLP-C - compatible with OLP; OLL - oral lichenoid lesions. * Pearson chi-square.

Symptoms, including mostly oral soreness, burning sensation and pain, were reported by 48.8% of the patients (n=160) and were more common in females (55%) than males (33%) (p=0.009). Symptoms were more frequently reported when there was involvement of the gingiva and by patients affected by atrophic/erosive/ulcerated lesions (associated or not to reticular areas) (p<0.0001). There were no statistically significant differences on the presence of symptoms when comparing the three groups.

Buccal mucosa was the most frequently affected site (157 cases, 71%), followed by the tongue (100 cases, 45.2%) and gingiva (44 cases, 19.9%). Buccal mucosa was the most common affected site for both females (114 patients, 71%) and males (43 patients, 70%); tongue was affected in 63 females (39%) in comparison to 37 males (61%) (p=0.004); and gingiva was affected in 38 females (24%) in comparison to 6 males (10%) (p=0.021). Buccal mucosa and tongue involvement was more frequent in OLP and gingival involvement was uncommon in OLL. The number of affected sites was higher in OLP and OLP-C groups in comparison to OLL, but the differences were not statistically significant (Table 1).

The reticular form alone was the most common clinical presentation (117 cases, 52.9%), followed by the association of reticular and atrophic/erosive/ulcerated lesions (92 cases, 41.6%) and atrophic/erosive/ulcerated lesions alone (12 cases, 5.4%). Plaque-like lesions were present in 11 cases (5%) and bulla were present in two cases (0.9%), but always associated with other clinical subtypes. The reticular pattern alone was more frequently found in OLP, while the association of reticular and atrophic/erosive/ulcerated patterns was the most common clinical presentation in OLP-C and OLL (p=0.025) (Table 1).

Patients reported a mean onset of the disease of 19.7 months (ranging from one to 360 months). Mean onset reported by females (22.6 months) was two times that reported by males (11 months) (p=0.261). Asymptomatic patients reported a longer onset interval (24.5 months) in comparison to symptomatic patients (16 months) (p=0.399). Similarly, patients affected exclusively by reticular lesions (21.5 months) reported a longer onset interval in comparison with patients affected by reticular and atrophic/erosive/ulcerated lesions (16.9 months) (p=0.628). There were no statistically significant differences on mean time of onset reported by OLP- (11.6 months), OLP-C- (22.3 months) and OLL-affected patients (45.8 months).

## Discussion

The clinicopathological features that precisely define OLP and OLL are still a matter of controversy. Although many studies have been published on this subject, there are several knowledge gaps in both clinical and histological parameters used to precisely differentiate both conditions. It has been previously shown that there is considerable clinical and histological intra and interobserver variability when analyzing lichenoid conditions ^6^ ^,^ ^11^. The most important limitations when comparing results derived from different studies are the inclusion and exclusion criteria used to select the sample in each individual study. In the present study we tried to strictly follow the histological criteria firstly suggested by the World Health Organization ^7^ and revised and modified by Van der Meij and van der Waal ^6^ and Cheng et al. ^2^ and included only cases that fullfilled them. Some previously published studies have included cases with no histologically proved lichenoid features in all cases ^12^ ^,^ ^13^, and/or including cases presenting epithelial dysplasia ^10^ ^,^ ^14^, possibly bringing some biases to data comparison. As the aim of the present study was to provide an oral pathologist´s perspective on this issue, we have intentionally included one group of patients (OLP-C) with histological features showing a lichenoid pattern without fullfilling all microscopic criteria for OLP. This OLP-C group is an important part of any Oral Pathology laboratory routine and deserves a specific analysis in order to understand its proximity (or not) to OLP. In this OLP-C group, the most common histological features that precluded interpretation of the sections as OLP were discontinuity of the band-like infiltrate and, consequently, of the basal cell liquefaction and the presence of focal sporadic deep chronic infiltrate.

Most published studies have included solely OLP patients and, consequently, few information is available on OLL and on the cases that do not completely fullfill the criteria for these two groups. Therefore, we have focused the present study on a comparison of the clinical features of specimens histologically interpreted as OLP, OLP-C and OLL in a sample of Brazilian patients. One limitation of the present study was that management and follow-up information of the patients were not available and information on behavior of these entities could not be retrieved from this sample.

Females were more affected than males in the present general sample and in each individual group. This predilection has been also demonstrated in almost all previously published studies on OLP, with predilection ranges varying from 61% to 80% ^8^ ^,^ ^10^ ^,^ ^11^ ^,^ ^12^ ^,^ ^13^ ^,^ ^14^ ^,^ ^15^ ^,^ ^16^ ^,^ ^17^ ^,^ ^18^ ^,^ ^19^ ^,^ ^20^ ^,^ ^21^ ^,^ ^22^ ^,^ ^23^ ^,^ ^24^ ^,^ ^25^, and also in OLL ^24^. Most patients are diagnosed on their fifties to sixties, with mean ages ranging from 50 to 67 years ^8^ ^,^ ^10^ ^,^ ^11^ ^,^ ^13^ ^,^ ^15^ ^,^ ^16^ ^,^ ^17^ ^,^ ^18^ ^,^ ^19^ ^,^ ^21^ ^,^ ^22^ ^,^ ^25^, but some studies including Asian ^14^ ^,^ ^20^, and also Brazilian ^23^ populations, have reported mean age of the affected patients in the fourties. It is interesting to notice that, similarly to our results, Mravak-Stipetic et al. ^11^ have reported that OLL usually affects older patients and do not show a marked predilection for females. In contrast, Aminzadeh et al. ^24^ have shown that OLL is more frequent in females and has a similar mean age than OLP. Feldmeyer et al. ^8^ and Aguirre-Urizar et al. ^10^ have reported a similar gender and age profile from the OLP and OLL patients included in their studies.

Symptoms are reported by 40 to 95% of the patients affected by OLP and the most common are oral soreness, burning sensation and pain ^12^ ^,^ ^14^ ^,^ ^15^ ^,^ ^16^ ^,^ ^18^ ^,^ ^19^ ^,^ ^21^ ^,^ ^23^ ^,^ ^25^. The presence of symptoms seems to be more frequent in females ^12^, is associated with the presence of atrophic/erosive/ulcerated lesions ^13^ ^,^ ^16^ ^,^ ^18^ ^,^ ^21^ ^,^ ^22^ ^,^ ^23^, is more common in more extensive lesions ^15^ and is not associated with any specific group (OLP, OLP-C and OLL), as also shown by the present results.

Clinical aspect of OLP, OLP-C and OLL can be quite similar and frequently brings additional diagnostic difficulties. Moreover, the concomitant presence of white (reticular, papular, and plaque-like) and red (atrophic, erosive, ulcerated and bullous) lesions is found in about 60% of the patients. The reticular pattern was the most common disease presentation in the present general sample and reticular lesions are the most commonly reported clinical pattern in OLP-affected patients as well ^8^ ^,^ ^12^ ^,^ ^13^ ^,^ ^14^ ^,^ ^18^ ^,^ ^20^ ^,^ ^22^ ^,^ ^23^, as also shown by our results. In contrast, in OLP-C- and OLL-affected patients the association of reticular and atrophic/erosive/ulcerated lesions was more common. Some studies, however, have shown that atrophic/erosive/ulcerated lesions were more common in their OLP patients ^10^ ^,^ ^15^ ^,^ ^16^ ^,^ ^17^ ^,^ ^19^ ^,^ ^21^ ^,^ ^25^. Feldmeyer et al. ^8^ have additionally shown that clinical aspect of the lesions in their OLL and OLP patients was similar. Although most studies did not depict any clinical differences in the distribution of the clinical pattern of the lesions in OLP ^12^, others have shown that the presence of the atrophic/erosive/ulcerated lesions is more common in females ^13^ ^,^ ^15^ ^,^ ^16^ ^,^ ^19^; that mean age of the patients affected by the atrophic/erosive/ulcerated pattern is older ^16^ ^,^ ^19^ ^,^ ^22^; that atrophic/erosive/ulcerated lesions are more common in the tongue and gingiva ^15^ ^,^ ^19^; and that atrophic/erosive/ulcerated are more extensive and/or multifocal ^15^ ^,^ ^22^. Mean age of the patients affected by reticular lesions and by the association reticular-atrophic/erosive lesions was similar in the present study, supporting that clinical presentation does not change over time from reticular to the atrophic-erosive-ulcerated pattern.

The most common intraoral locations for lichenoid conditions are the buccal mucosa (64 to 95% of the cases), tongue (especially the dorsum and lateral borders - 6 to 61%) and the gingiva/alveolar mucosa (3 to 68%) ^10^ ^,^ ^12^ ^,^ ^13^ ^,^ ^14^ ^,^ ^15^ ^,^ ^16^ ^,^ ^18^ ^,^ ^25^. The present results also showed that lichenoid conditions in general were more common in the buccal mucosa, tongue and gingiva; moreover, there were differences in the frequency of involvement of the tongue and gingiva when comparing females and males. In contrast, other authors have shown that there are no differences on site distribution according with gender in OLP ^12^. In the current series, there was a tendency that OLP-affected patients presented involvement of the buccal mucosa and tongue more frequently than the other two groups and that gingival involvement was less common in OLL. Aminzadeh et al. ^24^ showed that OLP affected mostly the buccal mucosa, followed by the gingiva and the tongue, while OLL showed a predilection for the buccal mucosa, but followed by the lips and tongue. Feldmeyer et al. ^8^ have shown that involvement of the buccal mucosa, dorsum of tongue and lips is less common in OLL than in OLP. It seems that, apart from the predilection for the buccal mucosa in both groups, OLP affects the tongue and gingiva more frequently and OLL has a predilection for the lips and tongue, being uncommon in the gingiva.

Most patients included in the present sample showed one (54%) or two concomitant affected sites (36%). Some studies focusing on OLP patients have shown that most cases presented lesions affecting more than one anatomical location in a bilateral and symmetric pattern (13-16). Patients from the OLP group presented lesions in more than one location (55%) more commonly than patients from the other two groups, but the difference was not statistically significant. The presence of symptoms was not associated with localized or widespread distribution of the oral lesions in the present sample.

Our results showed that the mean time of onset reported by the patients with lichenoid conditions was almost 20 months. Time of complaint was longer in females, in asymptomatic patients and in patients presenting reticular lesions alone, although none of these differences was statistically significant. Bagán-Sebastián et al. ^15^ reported that more extensive OLP lesions showed a longer time of onset. There were no differences when comparing time of complaint in OLP, OLP-C and OLL groups in the present study. Some authors, in contrast, reported that atrophic/erosive lesions were present for a longer period in comparison with reticular lesions ^22^.

The present study have selected all biopsies interpreted as lichenoid mucositis and separated them in three groups (OLP, OLL and OLP-C) according with the previously defined criteria. Clinical information was compared in the three groups and showed that, as expected, clinical features were different among them and helped to sustain the interpretation of the histological features. Moreover, specimens diagnosed as OLP-C showed clinical parameters close to OLP and availability of more clinical information and, eventually, indication of a biopsy in another clinically representative area would confirm diagnosis.

In conclusion, from the perspective of the oral pathologist, differences on gender and mean age of the affected patients, and anatomical location and clinical manifestation of the disease, would be helpful in characterizing OLP and OLL.
